# How to Measure “Short-Term Hormonal Effects”?

**DOI:** 10.1155/2009/459485

**Published:** 2009-09-29

**Authors:** Lothar A. J. Heinemann

**Affiliations:** Centre for Epidemiology & Health Research Berlin ZEG, Invalidenstrasse 115, D-10115 Berlin, Germany

## Abstract

*Background*. Interest to assess short-term benefits or risks of sex-steroid hormone use (OC or HRT) exists for years. However, no validated scale is available to evaluate the broad array of described effects of short-term hormone use. *Methods*. A raw scale consisting of 43 specific items and 47 general data was developed. Surveys in Italy, Germany and Austria were performed and data analyzed by factorial analyses. The resulting new scale with 15 items underwent reliability and validity investigations. *Results*. The new scale consists of 15 items in 5 domains. Internal consistency reliability coefficients were satisfactory as were test-retest reliability coefficients. Content and concurrent validity were promising. *Conclusion*. Psychometric properties of the new scale suggest good characteristics to measure short-term effects of sex-steroid hormones in women. The scale seems to be appropriate, feasible, interpretable, reliable, and valid for their application as PRO scale.

## 1. Introduction

For years, there has been an interest to evaluate short-term benefits or risks of sex-steroid hormone use in women, and many studies using nonvalidated scales were published. No validated scale was available that met the methodological guidelines for patient-reported outcome scales [1], that is, meeting the “state-of-the-art” psychometric requirements relevant for health-related quality-of-life (HRQoL) scales. 

 A variety of shorter or longer simple symptom lists were developed and applied in clinical studies, that is, only *subjectively *comparing the situation before and after hormone treatment in women. Such lists covered a broad array of clinically discussed short-term effect of hormone use such as skin and hair effects, effects on breast, on menstrual cycle/bleeding pattern, on aspects of sexual life, on psychological problems/symptoms, and on vegetative complaints associated with sex hormone use which will be discussed later. 

 The aim of this paper is to clarify if a validated measurement scale for short-term use of sex hormones can be developed and if yes, how the diagnostic characteristics such as reliability and validity of measurement might be. 

## 2. Material and Methods

Pertinent literature was scrutinized to get suggestions about potential self-perceived short-term benefits or adverse effects associated with the use of sex-steroid hormones such as sexual dysfunction particular during menopausal transition, problems of menstrual bleeding in general, hair and skin problems including breast tension/pain, and increasing body weight and also positive effects of hormones on premenstrual syndrome complaints were reported. Relief of menorrhagia, shortening of duration of bleeding [2], or treatment of dysfunctional uterine bleeding [3] including improvement of iron deficiency are commonly accepted benefits. The effects of controlling acne in combination drugs with certain progestagens [4, 5], or improvement of seborrhea [6, 7], were documented. More debates were found about hormonal effect on bleeding cycle-related disturbances [8–10]. 

 In addition, standardized HRQoL and other scales formerly used in clinical studies related to sex hormone use were reviewed to complete the array of interesting items potentially related with hormone use. This included the Menopause Rating Scale [11], the Quality of Sexual Function Scale, [12] the Female Sexual Function Index (FSFI) [13], the Derogates Interview for Sexual Functioning (DISF) [14], and the Female Sexual Distress Scale (FSDS) [15]. This included also experience gathered in studies with the Aging Males Symptoms Scale (AMS) [16]. 

 Own observational (prevalence) surveys in women (patients) with/without hormone use identified many differences in complaints between treated and untreated women and thereby candidates for items of a new scale (unpublished report) as well as a large, observational study in 9 countries in 4 continents [17] contributed also to the identification of possible problems or concerns of women (unpublished report). 

 The preparatory work resulted in a conceptual framework with five arbitrarily defined groups of items that could be relevant for a new validated scale (Table 1): psychological complaints (*n* = 8), somato-vegetative (*n* = 5), menstrual disorders (*n* = 8), sexual items (*n* = 13), and complaints related to hormone-sensitive organs (*n* = 7). The resulting raw scale consisted of 43 specific items (suspected short-term effects of sex steroid hormones), and of 47 general information (medical and reproductive history, demographic data) needed for the interpretation and item reduction. 

 The format of the new scale was planned as paper-based scale. Response categories at a Likert scale from 1 (= no, never) to 5 (= yes, severe) describe the personally perceived severity (or intensity) of complaints (items). All specific items were phrased in a negative direction (complaints) following own experiences with the development of other HRQoL scales [11, 12, 16]. If an item is not relevant, for example, the question of problems concerning menstrual bleeding in case of absence of menstrual cycle, “0” (no, not applicable) can be checked. 

 Thus, the raw scale consisted of an introduction, two examples as how to answer the questionnaire correctly, and the 90 items to be answered. The English version of the raw scale underwent a linguistic and cultural adaptation into Italian and German languages. 

 The statistical analyses are based on factorial analysis (main component analysis), Cronbach's alpha coefficient for internal consistency reliability, and test-retest-reliability. The statistical package SPSS 10 was used.

## 3. Results and Discussion

### 3.1. Development

The initial normative survey (Italy) involved 228 women aged 15–65 years. This was a sample of the normal female population, that is, not only women using hormones. This approach was chosen to get standard or norm values of the female population. 

 In a few steps of factorial analysis the number of items could be reduced and the domains of the final scale with 15 items determined. 

 Five dimensions (domains) were found, as similarly predicted in the conceptual framework (Table 2): sexual problems (SEX), menstrual problems (MENS), hormonal effects (HORM), psychological problems (PSYCH), and abdominal complaints (ABDOM). Table 2 summarizes the findings. For easy recognition and interpretation, only factor loadings over 0.5 were displayed. 

 In an independent population survey in Germany (*n* = 108), the same 5 domains were observed with factor loadings very similar if not identical with the Italian sample (data not shown here).

This supports the notion that the new scale and its domains have a quite good *face or content validity *confirmed in two independent studies.

### 3.2. Reliability

The internal consistency reliability—measured with Cronbach's Alpha—was good (*r* = 0.74) for the total scale and acceptable for the five domains with one exception: 0.73 on PSYCH, 0.73 HORM, 0.78 for MENS, 0.81 SEX, but unsatisfactory for ABDOM(= 0.53). The latter needs further research. The results were confirmed in the German validation study. 

 Additional information provided the item-domain correlation that showed strong associations of all items with the respective domains, however, with one exception: the items “cyclic bleeding from guts or bladder” with the domain “abdominal complaints” with a coefficient of 0.39. 

 Another aspect of reliability, test-retest reliability, was tested in a validation survey in Germany (*n* = 108): a very good reliability was observed for the total score (*r* = 0.92), and for the five domain scores: 0.83 for PSYCH, 0.85 for HORM, 0.93 for MENS, 0.72 for SEX, but unsatisfactory for ABDOM(= 0.62). The correlation coefficients were also good across almost all items of the scale. The test-retest reliability study confirmed what has been shown in the two studies with analyses of Cronbach's alpha.

### 3.3. Validity

The first step of validation is the comparability of the *internal structure* (dimensions) of a new scale throughout independent factor analyses and compatibility with the conceptual framework. This is indicative with a good face- or content-validity. 

 Since the SHE scale was designed also as health-related QoL scale with specific focus on short-term hormonal effects, we were particularly interested in evidence that the SHE scale really measures quality of life: the *SHE total score* significantly correlated with the generic QoL scale **SF-12** (total, physical, and mental health score) [18] in the German survey and similarly in an Austrian sample. The correlation coefficients were significant but not high, that is, ranging between *r* = 0.3 and *r* = 0.8). 

 Other significant but low correlations were observed between domains of the SHE-scale and the domain anxiety of the *HADS* [19] as well as with the domain psychosomatic QoL of the *QSF* in the German survey (ranging between *r* = 0.3 and *r* = 0.6). 

 The *psychological domain* of the SHE scale showed—as the total score—significant association with mental health and total score of SF-12, anxiety (HADS), and psychosomatic QoL (QSF) (range: *r* = 0.3–*r* = 0.6). The *hormone-related domain* showed correlations with SF-12 as well as QSF (range: *r* = 0.4–*r* = 0.5). The highest correlation of the *abdominal domain* of SHE was observed with SF-12 (total and physical health domain) (*r* = 0.6). The *menstrual domain* showed only a weak correlation with SF-12. The *sexual domain* score was correlated with anxiety (HADS) and with psychological QoL (QSF) (range *r* = 0.3 to *r* = 0.4). 

 Altogether, the SHE scale with its total and domain scores seem to be correlated with other scales intending to measure a similar content. 

### 3.4. Ability to Detect Changes

Since the SHE scale was not yet applied in treatment-related observational or the randomized clinical studies, there are no data to describe responsiveness or MID. 

 The next step will be an analysis of the sensitivity of the SHE scale to detect the effects of hormonal treatment. Therefore, the SHE scale should be included in relevant observational treatment studies or randomized clinical trials, that is, including also independent outcome variables for validation in the study. 

 The complete design/wording of the SHE scale (in German, English, Italian languages), the evaluation procedure, and reference (norm) values from the Italian, German, and Austrian population sample is openly accessible in the official website for the SHE scale (http://www.short-term-hormone-effects-scale.info). The scale can be used free of charge.

## 4. Conclusions

The newly developed SHE scale could close a gap for clinical research to measure short-term effects of sex-steroid hormones in women that were widely applied to demonstrate differences between relevant drugs. In the past, however, simple symptom lists based on the retrospective perception of an “improvement of conditions/complaints after therapy” or other not validated instruments were used as argument that specific formulations of sex-steroid hormones are better than others. Such not validated questionnaires lead to unreliable “benefits.” Although until now no validated scale was available meeting the FDA requirements for PRO scales, there is great interest of the industry to demonstrate “additional short-term benefits” of a newly developed drug containing sex steroid hormones in women because so many drugs are already on the market. 

 The assessment of the properties of the SHE scale is indicative of good characteristics to measure short-term effects of sex-steroid hormones in women. The scale seems to be appropriate, feasible, interpretable, reliable, and valid for their application as PRO scale. Data to assess responsiveness and sensitivity of the scale as outcome measure of hormone treatment are still lacking. 

 This validated scale can be recommended for practical use in comparative studies in order to avoid misjudgment concerning “benefits” provided by nonvalidated symptom lists with subjectively perceived “improvement” of drug A over drug B. As self-administered scale, the self-completion of the 15-item-scale takes less than 7 minutes on average. 

## Figures and Tables

**Table 1:** Conceptual framework and grouping of the complaints or symptoms possibly relevant for measuring short-term effects of hormones (raw scale). These “multicausal symptoms” were arbitrarily allocated to one of the five groups, that is, the groups are not mutually exclusive.

	Groups where symptoms or complaints might be related to				
	Psychological complaints	Somato-vegetative complaints	Menstrual, abdominal disturbances	Sexually related symptoms	Problems with hormone-sensitive Organs

Total number of symptoms	10	5	8	13	7

Listing of items of the raw scale	Reduced general well-being	Episodes of sudden sweating	Abdominal boating or swelling	Reduced sexual desire/libido	Breast tenderness or pain
	Depressive mood	Increased appetite, food craving	Abdominal pain	Reduced enjoyment of books or movies associated with sex	Temporary weight gain
	Sensation of loneliness	Nausea, dizziness	Cramps of guts or bladder	Less sexual fantasies/thoughts	Swelling of extremities
	Easily anxious	Joint and muscular discomfort	Cyclic bleeding from guts or bladder	Sexual life less enjoyable/satisfying	Greasy skin or acne
	Feeling worried	Heart discomfort	Irregular menstrual bleeding	Less satisfaction/enjoyment with sexual act	Hair disorders or problems
	Restlessness or irritability		Strong menstrual bleeding	Reduced vaginal lubrication during sex	Unexpected growth of body or facial hair
	Physical exhaustion		Painful menstrual period	Reduced sexual arousal	Suffering from cellulite
	Impaired capacity to concentrate		Significant premenstrual complaints that improve when menstrual flow began	Less sexual activity	
	Impaired memory			Lower frequency of orgasm	
	Sleep problems			Less satisfaction with orgasm	
				Pain during or after sex	
				Bladder problems	
				Dryness or burning vagina	

**Table 2:** Internal structure of the “Short-term Hormonal Effects (SHEs)” scale (factorial analysis; Italian normative survey; *n* = 228). The analysis determined five domains (explaining 66.1% of the total variance). The numbers in the table are factor loadings. Only loadings over 0.5 are shown.

	Factors/Domains				
Items	(1) SEX	(2) MENS	(3) HORM	(4) PSYCH	(5) Abdom

(1) Depressive mood				0.76	
(2) Feeling worried				0.83	
(3) Restlessness or irritability				0.77	
(4) Increased appetite, food cravings			0.67		
(5) Temporary weight gain			0.80		
(6) Swelling of extremities			0.81		
(7) Painful menstrual period		0.85			
(8) Significant premenstrual complaints that improve when the menstrual flow began		0.80			
(9) Strong menstrual bleeding		0.81			
(10) Less sexual fantasies/thoughts	0.83				
(11) Less satisfaction/enjoyment with sexual act	0.83				
(12) Reduced sexual arousal	0.82				
(13) Abdominal pain					0.61
(14) Cyclic bleedings from guts or bladder					0.79
(15) Cramps of guts or bladder					0.50

## References

[1] FDA—Guidance for Industry Patient-Reported Outcome Measures: Use of Medical Product Development to Support Labelling Claims. http://www.fda.gov/cder/guidance/5460dft.pdf.

[2] Larsson G, Milsom I, Lindstedt G, Rybo G (1992). The influence of a low-dose combined oral contraceptive on menstrual blood loss and iron status. *Contraception*.

[3] Davis A, Godwin A, Lippman J, Olson W, Kafrissen M (2000). Triphasic norgestimate-ethinyl estradiol for treating dysfunctional uterine bleeding. *Obstetrics and Gynecology*.

[4] Arowojolu AO, Gallo MF, Grimes DA, Garner SE (2004). Combined oral contraceptive pills for treatment of acne. *The Cochrane Database of Systematic Reviews*.

[5] Thorneycroft H, Gollnick H, Schellschmidt I (2004). Superiority of a combined contraceptive containing drospirenone to a triphasic preparation containing norgestimate in acne treatment. *Cutis*.

[6] Huber J, Foidart JM, Wuttke W (2000). Efficacy and tolerability of a monophasic oral contraceptive containing ethinylestradiol and drospirenone. *European Journal of Contraception and Reproductive Health Care*.

[7] Worret I, Arp W, Zahradnik H-P, Andreas J-O, Binder N (2001). Acne resolution rates: results of a single-blind, randomized, controlled, parallel phase III trial with EE/CMA (Belara) and EE/LNG (Microgynon). *Dermatology*.

[8] Joffe H, Cohen LS, Harlow BL (2003). Impact of oral contraceptive pill use on premenstrual mood: predictors of improvement and deterioration. *American Journal of Obstetrics and Gynecology*.

[9] Parsey KS, Pong A (2000). An open-label, multicenter study to evaluate Yasmin, a low-dose combination oral contraceptive containing drospirenone, a new progestogen. *Contraception*.

[10] Sangthawan M, Taneepanichskul S (2005). A comparative study of monophasic oral contraceptives containing either drospirenone 3 mg or levonorgestrel 150 *μ*g on premenstrual symptoms. *Contraception*.

[11] Heinemann K, Ruebig A, Potthoff  P (2004). The menopause rating scale: a methodological review. *Health and Quality of Life Outcomes*.

[12] Heinemann LAJ, Potthoff P, Heinemann K, Pauls A, Ahlers CJ, Saad F (2005). Scale for Quality of Sexual Function (QSF) as an outcome measure for both genders?. *Journal of Sexual Medicine*.

[13] Rosen R, Brown C, Heiman J (2000). The Female Sexual Function Index (FSFI): a multidimensional self-report instrument for the assessment of female sexual function. *Journal of Sex and Marital Therapy*.

[14] Derogates LR (1997). The Derogates Interview for Sexual Functioning (DISF)/DISF-R): an introductory report. *Journal of Sex and Marital Therapy*.

[15] Rosen RC (2002). Assessment of female sexual dysfunction: review of validated methods. *Fertility and Sterility*.

[16] Daig I, Heinemann LAJ, Kim S (2003). The Aging Males' Symptoms (AMS) scale: review of its methodological characteristics. *Health and Quality of Life Outcomes*.

[17] Heinemann K, Rübig A, Strothmann A, Nahum GG, Heinemann LAJ (2008). Prevalence and opinions of hormone therapy prior to the women's health initiative: a multinational survey on four continents. *Journal of Women's Health*.

[18] Ware JE, Kosinski M, Keller SD (1996). A 12-item short-form health survey: construction of scales and preliminary tests of reliability and validity. *Medical Care*.

[19] Zigmond AS, Snaith RP (1983). The hospital anxiety and depression scale. *Acta Psychiatrica Scandinavica*.

